# Update on the treatment of metastatic clear cell and non-clear cell renal cell carcinoma

**DOI:** 10.1186/s40364-015-0030-7

**Published:** 2015-03-02

**Authors:** Kevin Y Xu, Shenhong Wu

**Affiliations:** Department of Medical Education, Icahn School of Medicine at Mount Sinai, New York, NY USA; Division of Hematology/Oncology, Department of Medicine, Stony Brook University School of Medicine, Room: HSC 15-053E, Stony Brook, NY USA; Northport VA Medical Center, Northport, NY USA

**Keywords:** Renal cell carcinoma, Clear cell, Non-clear cell, PD-1, Tyrosine kinase inhibitor

## Abstract

The advent of new knowledge surrounding the molecular pathology of renal-cell carcinoma (RCC) has culminated in a number of emerging targeted therapies. In just the last year, several new studies have been able to translate our understanding of tumor biology into significantly improved outcomes in patients with advanced RCC. The objective of this review is to describe new developments in targeted treatments and immunotherapies for patients with both clear-cell and non-clear cell metastatic RCC following the 2014 American Society of Clinical Oncology (ASCO) annual conference. We will discuss new applications of anti-VGF agents and PD-1 inhibitors in order to shed light on emerging avenues of RCC treatment that show considerable promise.

## Introduction

With the development of multiple anti-VEGF (vascular endothelial growth factor) agents and mTOR (mammalian target of rapamycin) inhibitors, we have seen significant progress over the last few years in targeted therapies for treating renal cell carcinoma (RCC). While these targeted therapies were largely tested in patients with the more common clear cell RCC (ccRCC), recent studies have begun to investigate the efficacy of targeted therapy in the much less common, non-clear cell RCC (nccRCC). In addition, a number of new studies have, for the first time, examined the activity of new immunotherapies in treating ccRCC. In this article, we performed a brief review of recent studies on the treatment of RCC with an update from 2014 annual ASCO meeting.

## Review

RCCs are characterized by a vast array of different histological and cytogenetic signatures. Of approximately 64,000 cancers of the kidney every year [1], 80% are clear cell renal cell carcinoma (ccRCC) and 20% are non–clear cell renal cell carcinoma (nccRCC) subtypes.

VEGF signaling plays a significant role in tumorigenesis in light of its role in angiogenesis, vascular permeability, and cancer stem cell development. It is important to note that the more common ccRCC has often been tied to the occurrence of von Hippel-Lindau (VHL) mutations implicated in VEGF signaling deregulation [2]. Such mutations have been found to contribute to an accumulation of transcription factor HIF1α (hypoxia-inducible factor 1-alpha) and, consequently, increased levels of VEGF and other growth factors. Because the up-regulation of VEGF activity has corresponded to the uncontrolled modulation of the hypoxic response, multiple agents (e.g., sorafenib, sunitinib, bevacizumab, pazopanib, axitinib, temsirolimus, everolimus) have been identified as abrogators of VEGF-mediated signaling [3,4]. These therapeutic agents operate through a variety of mechanisms, many of which abrogate specific components of the VEGF signaling (TKIs for VEGFR, bevacizumab for VEGF).

In addition to VEGF pathway deregulation, mTOR kinase is a well-known contributor to tumorigenesis. Given that a number of downstream mTOR effectors regulating angiogenesis, metabolism, and cell growth have been found to be deregulated in cancers, various targeted therapies such as temsirolimus and everolimus have been developed to hinder mTOR signaling. These two mTOR signaling-based therapies will be discussed in upcoming paragraphs.

### Targeted therapy for RCC with bone metastases

In recent years, a considerable number of tyrosine-kinase inhibitors (TKIs) have been developed that seek to target VEGF signaling in ccRCC. Among the most prevalent of such targeted ccRCC therapies are TKIs such as pazopanib, sorafenib, and axitinib, which have been found to lead to significantly improved outcomes for metastatic renal cell carcinoma (mRCC) patients. For instance, bevacizumab, a humanized anti-VEGF antibody, was found in phase III randomized controlled trials (RCTs) to contribute to significant increases in progression-free survival in mRCC patients who had used interferon alfa as first-line treatment [5]. An oral angiogenesis inhibitor (pazopanib) targeting VEGFR, PDGFR (platelet-derived growth factor receptor), and the c-kit tyrosine kinase were found to demonstrate significant improvements in progression-free survival and tumor response in both treatment-naive and pre-treated patients with mRCC [6]. Paralleling pazopanib’s effects, sorafenib (an oral multikinase inhibitor of VEGF receptors, platelet-derived growth factor receptors, and Raf kinases) was found in a phase III RCT to contribute to significantly longer median progression-free survival in mRCC patients compared to placebo [7]. Building off the trial’s promising results, a more recent phase III RCT revealed that the second-generation inhibitor of VEGF receptors, axitinib, culminated in even longer progression-free survival (PFS) than sorafenib, supporting this VEGFR inhibitor’s suitability as a second-line therapies for mRCC [8]. Besides VEGF inhibition, mTOR inhibitors such as temsirolimus (an inhibitor of rapamycin kinase) have also been found to significantly improve overall survival (OS) among mRCC patients with poor prognosis [9].

While the efficacy of TKIs in treating metastatic ccRCC has been well established, a new body of research has sought to examine the therapeutic value of TKIs in treating mRCC patients with bone metastases (BM), long considered one of the most destructive of mRCC complications. Despite the high prevalence of BM, therapies for BM management have been very limited in advanced renal cell carcinoma in light of the poorer prognosis that patients with BM have relative to those who do not have BM. In a retrospective review of 375 mRCC patients presented in this year’s ASCO annual meeting, the median survival of BM patients using TKI was found to be significantly longer than those who had not used TKIs [22 months (95% CI: 17–25) vs. 14 months (95% CI: 10–19), p < 0.01] [10]. However, mRCC patients with BM who were treated with TKIs were observed to have a similar median OS as patients without BM (p = 0.66). It necessitates emphasis that this study was limited by its retrospective nature, lack of randomization or blinding, and accuracy of documentation. Future prospective clinical trials may be warranted to better understand the interplay between different TKIs and their efficacy on mRCC patients with BM.

### New immunotherapy (PD-1 inhibitors) for clear cell RCC

Besides targeted therapies, immunotherapies seeking to break tumor tolerance are another area of interest in the RCC research community. Instead of directly targeting malignant cells and stroma, immunotherapies work to up-regulate the host immune response in order to destroy neoplastic cells that escape immune recognition.

An immune signaling mechanism of particular interest to cancer clinicians is the PD-1 pathway. A potent immune checkpoint receptor, programmed death-1 (PD-1) is a cell-surface glycoprotein on T cells, B cells, and macrophages that inhibits their activation. In recent years, therapeutic agents inhibiting the PD-1 pathway and increasing the strength of the host’s anti-tumor immune response have been found to show therapeutic efficacy in the treatment of various cancers. This past year, for instance, the PD-1 agent pembrolizumab (MK-3475) was found to contribute to a 1-year survival rate of 69% in metastatic melanoma patients with prior systematic therapies of ipilimumab, leading to its FDA approval for use in second-line settings [11]. In a separate PD-1 inhibitor trial, the monoclonal antibody nivolumab (used in combination with ipilimumab) was found to produce a 1-year 85% survival rate and 2-year 79% survival rate in advanced melanoma patients [12]. In another phase 1 study, this time on patients with metastatic renal cell carcinoma, nivolumab and ipilimumab were found to contribute to a response rate of 43% (N3 + I1 arm; nivolumab at 3 mg/kg and ipilimumab at 1 mg/kg) and 48%. (N1 + I3 arm; nivolumab at 1 mg/kg and ipilimumab at 3 mg/kg). The aforementioned study’s results were encouraging, for the N3 + I1 arm was found to have a median progression-free survival of 36.6 weeks, whereas the N1 + I3 arm had a median progression free survival of 38.3 weeks [13].

Mirroring their efficacy in treating melanoma, PD-1 inhibitors have also been found to be potent agents in the treatment of RCC. Given that the PD-L1 ligand has been found to be correlated with poor mRCC prognosis, a number of PD-1 inhibitors (nivolumab, lambrolizumab, ZBMS-936559, MPDL3280A, AMP-224) are currently being investigated for their therapeutic efficacy [14]. In addition to its demonstrated therapeutic activity in treating melanoma, nivolumab has been found to have significant benefits in the treatment of mRCC. In a phase II study of nivolumab’s activity in clear-cell mRCC patients pretreated with anti-VEGR agents presented in ASCO 2014 annual meeting, researchers examined dose–response relationships, overall survival, objective response rate, and drug safety [15]. Patients were randomized and blinded to receive nivolumab at three different doses: at 0.3 mg/kg, 2 mg/kg, or 10 mg/kg. Although no dose–response relationship for progression-free survival (PFS) was found, significant anti-tumor activity was observed with the usage of nivolumab, including objective responses of relatively long duration. While the 0.3 mg/kg group had an overall survival (OS) of 18.2 months, median OS were not reached for other groups. Across doses, 19 out of 35 responders (54%) had objective responses lasting greater than 12-20+ months. Overall, nivolumab was considered to be well-tolerated, for less than 17% of patients (across all doses) experienced grade 3–4 adverse events [15].

Despite its demonstrated activity, there is limited biomarker data to predict the therapeutic effect provided by nivolumab in treating clear-cell mRCC. A recent study presented in ASCO 2014 annual meeting, however, has provided valuable insight on the molecular mechanisms underlying nivolumab’s activity by examining the serum of mRCC patients treated with nivolumab for chemokines and T cell infiltrates at baseline, day 8 of cycle 2 (biopsy), and day 2 of cycle 1 (serum) [16]. Employing a phase I open-label study design with four parallel treatment arms (1–3 for patients with previously treated mRCC, arm 4 for treatment-naïve nRCC patients), the Choueiri et al. research team accumulated data on overall response rate, safety/tolerability, and treatment-induced changes in PD-L1 expression. Evidenced by increases in interferon-gamma signaling, T-cell tumor infiltrates in biopsies, and serum concentrations of CXCL9 and CXCL10, nivolumab was found to have significant clinical activity in 91 patients with both mRCC previously treated with TKIs and treatment-naive mRCC. The researchers observed an increase in immune activity for both serum chemokines CXCL9 (191%) and CXCL10 (90%), as well as an increase in T cell infiltrates by 70% (CD3+) and 88% (CD8+). Changes in biomarkers were consistent with PD-1 inhibition and provided evidence of immunomodulatory effects in serum and in the tumor microenvironment. The objective response rate was 16% (16% in previously treated patients; 13% in untreated patients), and the median duration of response was 15 months. Responses were numerically higher in PD-L1^+^ patients but were also seen in PD-L1^−^ patients. The promising results of the Choueiri et al. trial will hopefully pave the way for future biomarker-based studies that examine links between therapeutic doses and the molecular activity of biological agents implicated in pharmacodynamics and cancer cell function.

### Targeted therapy for non-clear cell RCC

While the RCC therapies discussed above were for the treatment of clear-cell renal cell carcinoma, the optimal systemic therapy for non-clear cell renal cell carcinoma remains a topic of debate among clinicians. While the most common histological classification among nccRCCs is papillary renal cell carcinoma (10-15%), other histologies include chromophobes (5-10%), oncocytomas, renal medullary carcinomas, collecting-duct carcinomas, sarcomatoid, and unclassified nccRCCs. Given that VHL mutations do not play a role in the non-clear cell renal carcinoma disease course, it is unclear whether anti-angiogenic TKIs targeting VEGF–a standard of care in the treatment of clear-cell RCC–would have a therapeutic benefit to mRCC patients. In addition, the relative rarity of non-clear cell RCC in comparison to ccRCC poses a challenge to clinical researchers seeking to gain insights on a possible link between TKIs and non-clear cell RCC pathogenesis.

Despite these challenges, researchers have begun examining the novel application of VEGFR TKIs in the treatment of non-clear cell RCC and have found evidence suggestive that VEGF TKIs have therapeutic value in the treatment of nccRCC. Two drugs in particular, sunitinib and the mTOR inhibitor everolimus, have been investigated for their possible treatment benefits in nccRCC patients. In 2007, sunitinib, the aforementioned orally administered inhibitor of tyrosine kinases ranging from VEGFRs to PDGFRs, was found to contribute to significantly higher progression-free survival and response rate in mRCC patients participating in a phase III trial [17]. In the following year, another phase III trial led by the Motzer et al. group revealed that everolimus prolonged progression-free survival compared to placebo in a sample of mRCC patients after sorafenib and/or sunitinib treatment [18].

In a randomized phase 2 trial of non-clear cell RCC presented in the 2014 ASCO annual meeting (the ESPN trial), researchers sought to evaluate the assumption that mTOR inhibitors like everolimus may benefit “poor-risk” disease patients such as those with nccRCC in terms of improved progression-free survival and overall survival. Employing a crossover study design, researchers evaluated the premise that median PFS would be improved from 12 weeks with sunitinib to 20 weeks with everolimus. Amid a sample of 68 participants (27 papillary, 11 chromophobe, 9 unclassified, 7 translocated, 13 sarcomatoid, 1 oncocytic), researchers noted significantly higher overall survival in the sunitinib arm (not reached vs 10.5 months, p = 0.01). Chromophobe histology was also found to have better overall survival and progression-free survival than other histologies in both arms [19].

Because of the superior overall survival among participants taking sunitinib, the ESPN study was prematurely terminated as recommended by data and safety monitoring committee; differences in progression-free survival were consequently not observed in the first-line between sunitinib (6.1 months) and everolimus (4.1 months, p = 0.6). Following cross-over, the progression-free survival in the second line was relatively short: 1.8 months for sunitinib and 2.8 months for everolimus (p = 0.6). Based on futility analysis for progression-free survival and inferior overall survival with everolimus compared to sunitinib in the first-line setting, termination of further patient accrual was recommended for the trial, and consequently everolimus was not recommended as a first-line treatment option in nccRCC. It warrants emphasis that everolimus does not target mTOR complex 2, which has been identified as a powerful driver of tumor proliferation [20]. Given that the targeting of mTOR-2 could potentially contribute to RCC treatment benefits, future research is needed on the efficacy of therapies that target components of the mTOR signaling besides solely mTOR-1.

## Conclusions

In summary, the metastatic ccRCC treatment landscape–previously dominated by targeted therapies–could be altered significantly with the advent of potent immunotherapies that abrogate the PD-1 pathway. However, targeted therapies such as TKIs continue to hold much promise in the management of clear cell and nccRCC. Future randomized-controlled studies are needed in order to determine if new PD-1 inhibitors or their combination with TKIs may provide survival benefits in the treatment of RCC.

## Abbreviations

BM: Bone metastases
ccRCC: Clear cell renal cell carcinoma
HIF1-α: Hypoxia-inducible factor 1-alpha
mRCC: Metastatic renal cell carcinoma
mTOR: Mammalian target of rapamycin
nccRCC: Non-clear cell renal cell carcinoma
OS: Overall survival
PD-1: Programmed death-1
PDGFR: Platelet-derived growth factor receptor
PFS: Progression-free survival
RCC: Renal cell carcinoma
RCT: Randomized controlled trial
TKI: Tyrosine kinase inhibitor
VEGF: Vascular endothelial growth factor
VHL: Van-Hippel lindau
